# Development and validation of a Nomogram for the prediction of patients with sepsis-induced multiple organ dysfunction syndrome

**DOI:** 10.12669/pjms.41.4.10421

**Published:** 2025-04

**Authors:** Jinling Ji, Qiong Wang, Kai Wang, Ting Shi, Chang Li

**Affiliations:** 1 Jinling Ji, Department of Medical Laboratory, The Affiliated Huai’an No.1 People’s Hospital of Nanjing, Medical University, Huai’an, Jiangsu223300, China; 2 Qiong Wang, Department of Medical Laboratory, The Affiliated Huai’an No.1 People’s Hospital of Nanjing, Medical University, Huai’an, Jiangsu223300, China; 3 Kai Wang, Department of Rheumatology, The Affiliated Huai’an No.1 People’s Hospital of Nanjing, Medical University, Huai’an, Jiangsu223300, China; 4 Ting Shi, Department of Hepatobiliary and Pancreatic Surgery, The Affiliated Huai’an No.1 People’s Hospital of Nanjing, Medical University, Huai’an, Jiangsu223300, China; 5 Chang Li, Department of Medical Laboratory, The Affiliated Huai’an No.1 People’s Hospital of Nanjing, Medical University, Huai’an, Jiangsu223300, China

**Keywords:** Nomogram, Probability, Risk, Sepsis, SI-MODS, Variable

## Abstract

**Objective:**

To develop and validate a model capable of predicting the risk of Sepsis-induced multiple organ dysfunction syndrome (SI-MODS) in hospitalized sepsis patients.

**Methods:**

A retrospective cohort study was performed to analyze the clinical data of 415 patients admitted to Department of Medical Laboratory, The Affiliated Huai’an No.1 People’s Hospital of Nanjing between January 2019 and January 2022. The least absolute shrinkage and selection operator (LASSO) regression analysis was employed to pinpoint potential variables. A nomogram was developed through multivariate logistic regression. For internal validation, the bootstrapping method was utilized. The nomogram’s performance was assessed through calibration, discrimination, and clinical utility analyses.

**Results:**

Among the 415 patients, SI-MODS was identified in 46 individuals (11.1%). This model identified seven key variables. The model’s internal validation yielded an area under the curve of 0.903 (95% CI: 0.863-0.943). The model’s calibration was strong, and results from a decision curve analysis showed that the created nomogram provided a net benefit across a threshold probability range of 1–66% for predicting SI-MODS.

**Conclusion:**

Our study develops a nomogram incorporating based on PaO2, LAC, multidrug resistant bacteria, septic shock, coagulation disorder, mechanical ventilation, and kidney failure can predict the risk of MODS in sepsis patients, which helps clinicians make risk based decisions and treatment strategies.

## INTRODUCTION

Multiple organ dysfunction syndrome (MODS) is characterized as an acute and potentially reversible dysfunction affecting two or more organs, caused by various factors.^1^ Studies indicate that MODS affects 11-40% of adult patients admitted to intensive care units (ICUs).^2^,^3^ MODS is prevalent among critically ill patients and is associated with a high mortality rate of 44-76%, primarily due to lack of effective treatments. Consequently, early detection and intervention in the development of MODS are crucial for improving clinical outcomes.^4^

MODS is the most severe outcome of sepsis progression and is highly correlated with a worse prognosis.^5^ Recent efforts have been made in developing prediction models for sepsis-induced multiple organ dysfunction syndrome (SI-MODS). Benscoter et al. introduced a novel risk prediction model that assessed the risk of developing multiple organ dysfunction following pediatric cardiac surgery that required cardiopulmonary bypass (CPB). The optimal model, which includes interleukin-8 (IL-8), chemokine ligand 3 (CCL3), and age as predictor variables, achieved an area under the receiver operating characteristic curve (AUROC) of 0.75 through ten-fold cross-validation.^6^ It was demonstrated by Atreya et al, the utility of a supervised machine learning approach for identifying 20 genes, achieving AUROC ranging from 0.74 to 0.79 in validation and test sets for predicting persistent MODS across different ages and causes of organ dysfunction^7^ Given the suboptimal performance and limited interpretability of existing models, and the scarcity of research on predictive models for the early diagnosis of SI-MODS, there is a clear need for a new model.^8^

This study was designed to develop a nomogram model based on independent risk factors to predict the risk of MODS in sepsis patients.

## METHODS

From January 2019 and January 2022, patients diagnosed with sepsis were admitted to the Affiliated Huai’an No.1 People’s Hospital of Nanjing Medical University and subsequently enrolled in this retrospective cohort study using simple random sampling.

### Inclusion criteria:

- Diagnosis of sepsis according to the Sepsis 3.0 guidelines.^9^
- Age 18 years or older.
- A minimum intensive care unit (ICU) stay of 48 hours.
- At least one instance meeting the MODS diagnostic criteria. For patients with multiple ICU admissions during their hospital stay, only data from the initial admission were considered.

### Exclusion criteria:

- Patients younger than 18 years.
- Pregnant women, confirmed via blood or urinary pregnancy tests.
- Individuals with HIV infection and a known CD4 cell count of 200/mm^3 or less.
- Patients with active neoplasms or other medical conditions not related to sepsis.
- Neutropenia, defined as having fewer than 1000 neutrophils per cubic millimeter of blood.
- Patients who either died or were discharged within 24 hours after ICU admission.

### Ethical Approval:

The hospital’s ethical review board approved the study protocol (approval number: KY-2023-156-01, dated Sept. 6, 2023). Informed consent was waived as the data were anonymized, per ethical board approval. The procedures in this study were performed in accordance with the Helsinki Declaration of 1975.

### Data Collection:

We reviewed the clinical data of patients in the Electronic Medical Records Systems (EMRS) and Picture Archiving and Communication Systems (PACS). Data were collected retrospectively, encompassing demographic information, chronic complications, laboratory test, Simplified Acute Physiology Score II (SAPS-II) and Sequential Organ Failure Assessment (SOFA) scores and others.

### SI-MODS:

Data from all eligible sepsis patients were analyzed to identify those with MODS, defined according to the scoring system developed by Marshall et al.^10^ This scoring system incorporates clinical and laboratory variables across six organ systems. A total score is computed, as detailed in Table-I, which assists clinicians in predicting the mortality risk of critically ill patients.

**Table-I:** The multiple organ dysfunction score.

Organ system	0	1	2	3	4
Respiratory (PaO_2_/FIO_2_)	>300	226~300	151~225	76~150	≤75
Renal (serum creatinine)	≤100	101~200	201~350	351~500	>500
Hepatic (serum bilirubin)	≤20	20~61	61~120	121~240	>240
Cardiovascular (PAR)	≤10.0	10.1~15.0	15.1~20.0	20.1~30.0	>30
Hematologic (platelet count)	>120	81~120	51~80	21~50	≥20
Neurologic (Glasgow Coma Score)	15	13~14	10~12	7~9	≤6

*Abbreviations:* PaO_2_/FIO_2_: Arterial pressure of oxygen/inspiratory fraction of oxygen; PAR: Pressure-adjusted heart rate; RAP: Right atrial pressure.

### Statistical Analysis:

Continuous variables are summarized using the median and interquartile range and compared employing the Wilcoxon rank-sum test. Categorical variables are presented as numbers and percentages and are compared using either Chi-square tests or Fisher’s exact test. Feature selection is a critical step in constructing models. Predictor selection and model regularization were performed using the Least Absolute Shrinkage and Selection Operator (LASSO) regression analysis. Following this, a multivariate logistic regression analysis was conducted to develop a predictive model that distinguishes between SI-MODS and non-SI-MODS patients.

This model was then used to create a nomogram. The discriminative performance of the model was assessed by calculating the area under the receiver operating characteristic curve (AUC). For internal validation, a bootstrapping analysis with 500 iterations was conducted.^11^ The Hosmer-Lemeshow test was conducted to assess the calibration of the model. Additionally, Decision Curve Analysis (DCA) was performed to determine the clinical utility of the model.^12^ Data were statistically analyzed using the R software (v3.6.3). The significance level was established at *p* <0.05.

## RESULTS

**Fig.1:**
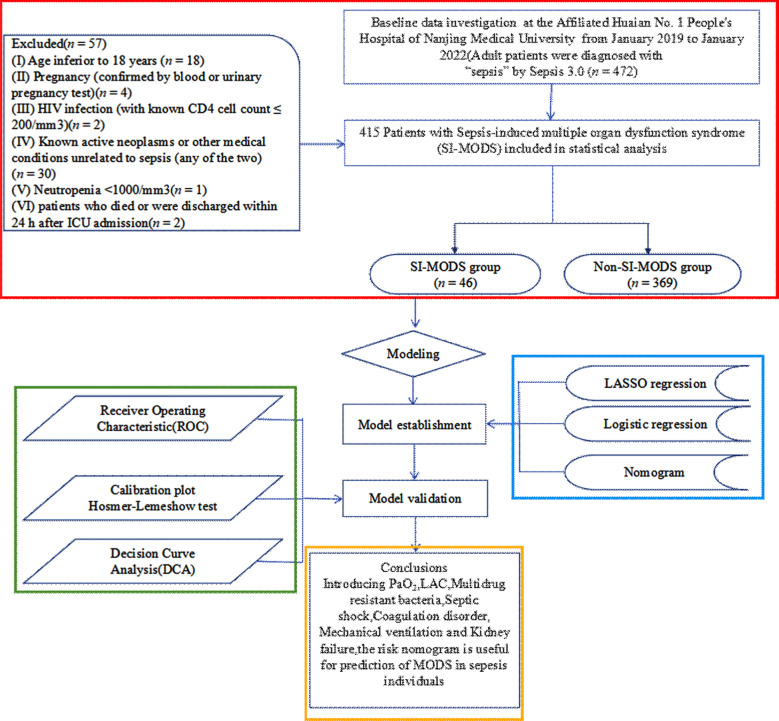
Flowchart of data extraction and study design. Abbreviations: PaO_2_: Arterial partial pressure of oxygen; LAC: Lactate. LASSO: Least absolute shrinkage and selection operator.

A total of 472 patients were diagnosed with sepsis upon admission according to Sepsis 3.0 criteria. Following exclusion criteria application, 57 patients were excluded as shown in Fig.1. Consequently, 415 patients were included in the study, among whom 46 (11.1%) developed SI-MODS after ICU admission. Table-II and III describes the differences in characteristics between the SI-MODS and non-SI-MODS groups.

**Table-II:** Demographic characteristics of the participants.

Variables	All patients (n = 415)	Non-SI-MODS patients (n = 369)	SI-MODS patients (n = 46)	Fisher/χ^2^	p-Value
*Demography*					
Gender, *n* (%)				0.705	0.401
Female	179 (43)	156 (42)	23 (50)		
Male	236 (57)	213 (58)	23 (50)		
Age, *n* (%)				1.406	0.236
<60 years	118 (28)	101 (27)	17 (37)		
≥60 years	297 (72)	268 (73)	29 (63)		
*Laboratory results*					
PT, (s)	15.6 (13.65, 17.7)	15.5 (13.6, 17.6)	16.05 (13.85, 23.13)	7160.5	0.084
INR	1.27 (1.12, 1.49)	1.27 (1.12, 1.48)	1.38 (1.16, 2.2)	6804	0.028
APTT, (s)	37.9 (31, 47.15)	38 (31.2, 47)	37.55 (30.15, 47.47)	8457.5	0.970
TT, (s)	16.9 (15.8, 18.6)	16.9 (15.8, 18.5)	17.7 (15.88, 20.27)	7359	0.142
FIB, (g/L)	4.34 (2.97, 5.91)	4.5 (3.19, 6.07)	3.5 (2.17, 4.58)	10962.5	0.001
D-dimer, (ug/ml)	4.42 (2.3, 7.94)	4.42 (2.25, 7.81)	4.44 (2.4, 11.76)	7926	0.465
PH,	7.42 (7.34, 7.47)	7.42 (7.35, 7.47)	7.41 (7.31, 7.46)	9686.5	0.118
PaCO_2_, (mmHg)	34.5 (29.55, 39.1)	34.5 (30, 39.1)	33.4 (28.05, 39.6)	8953.5	0.544
PaO_2_, (mmHg)	83.4 (64.25, 109)	85.5 (65.2, 109)	77.85 (63.37, 103.22)	9087	0.434
BE, (mmol/L)	-3 (-7.45, 1.8)	-3 (-6.6, 2.2)	-2.95 (-12.08, 0.5)	9597.5	0.148
HCO3, (mmol/L)	21.2 (16.9, 24.95)	21.2 (16.9, 25.6)	21.2 (18.05, 24.15)	8820.5	0.664
SO2, (%)	96.3 (92.85, 98.4)	96.3 (92.9, 98.3)	96.3 (92.8, 98.9)	8331	0.839
LAC, (mmol/L)	2.5 (1.6, 4.4)	2.4 (1.5, 4.3)	4.35 (2.3, 12.05)	5441.5	< 0.001
WBC, (K/uL)	10.57 (6.21, 17.12)	10.73 (6.37, 17.16)	9.36 (4.74, 14.31)	9662.5	0.126
Neutrophil, (K/uL)	9.29 (4.67, 15.34)	9.65 (4.8, 15.66)	7.57 (3.52, 13.05)	9829	0.08
Lymphocyte, (K/uL)	0.77 (0.46, 1.19)	0.77 (0.45, 1.15)	1 (0.55, 1.39)	7474	0.187
Platelet level, (K/uL)	141 (79.5, 214.5)	143 (84, 217)	121.5 (49.75, 193.25)	9955	0.056
Totalbilirubin, (µmol/L)	16.1 (9.9, 29.7)	15.5 (9.5, 28.5)	22.45 (12.75, 53.45)	6336.5	0.005
NT-proBNP, (pg/ml)	3063 (1106.5, 10683.5)	2979 (1040, 10113)	6119 (1854.75, 20460)	6815	0.029
Albumin, (g/L)	29.8 (25.85, 33.95)	29.8 (25.8, 34)	29.4 (26.22, 32.27)	8847	0.639
Creatinine, (µmol/L)	98 (66, 170.75)	98 (66, 170)	100.5 (66.85, 175.65)	8389.5	0.899
BUN, (µmol/L)	10.59 (6.85, 15.63)	10.25 (6.68, 15.32)	12.93 (7.89, 16.02)	7691	0.300
ALT, (IU/L)				Fisher	< 0.001
≤400	384 (93)	349 (95)	35 (76)		
> 400	31(7)	20 (5)	11 (24)		
AST, (IU/L)	43 (25, 105.05)	40 (23.2, 87)	107.65 (49.2, 424)	4611.5	< 0.001
GGT, (IU/L)	39 (18, 79)	38 (18, 78)	40 (19.25, 97)	8249	0.757
LDH, (IU/L)	290 (217, 449.5)	285 (212, 418)	464.5 (292.5, 1602.5)	5168.5	< 0.001
PCT, (ng/ml)	11.56 (1.58, 52.57)	11.18 (1.59, 55.42)	14.86 (1.2, 32.54)	8853	0.634

*Abbreviations*: SI-MODS: Sepsis-induced multiple organ dysfunction syndrome; PT: Prothrombin time; INR: International normalized ratio; APTT: Activated partial thromboplastin time; TT: Thrombin time; FIB: Fibrinogen; PaCO_2_: Arterial carbon dioxide tension; PaO_2_: Arterial partial pressure of oxygen; BE: Buffuer excess; HCO3: Bicarbonate; SO_2_: Sulfur dioxide; LAC: Lactate; WBC: White blood counts; NT-proBNP: N-terminal pro B-type natriuretic peptide; BUN: Blood urea nitrogen; ALT: Alanine aminotransferase; AST: Aspartate aminotransferase; GGT: Gamma-glutamyltransferase; LDH: Lactic dehydrogenase. PCT: Procalcitonin.

**Table-III:** Other data of the participants.

Variables	All patients (n = 415)	Non-SI-MODS patients (n = 369)	SI-MODS patients (n = 46)	Fisher/χ^2^	p-Value
Treatment					
Vasoactive drugs, *n* (%)				1.545	0.214
No	323 (78)	291 (79)	32 (70)		
Yes	92 (22)	78 (21)	14 (30)		
Cardiovascular drugs, *n* (%)				1.045	0.307
No	308(74)	271 (73)	37 (80)		
Yes	107 (26)	98 (27)	9 (20)		
Anticoagulants, *n* (%)				53.138	< 0.001
No	326 (79)	309 (84)	17 (37)		
Yes	89 (21)	60 (16)	29 (63)		
Tracheotomy, *n* (%)				Fisher	0.256
No	380 (92)	340 (92)	40 (87)		
Yes	35 (8)	29 (8)	6 (13)		
Mechanical ventilation, *n* (%)				18.535	< 0.001
No	140 (34)	138 (37)	2 (4)		
Yes	275 (66)	231 (63)	44 (96)		
Tracheal intubation, *n* (%)				1.526	0.217
No	247 (60)	224 (61)	23 (50)		
Yes	168 (40)	145 (39)	23 (50)		
Unable to wean off the ventilator, *n* (%)				2.758	0.097
No	316 (76)	286 (78)	30 (65)		
Yes	99 (24)	83 (22)	16 (35)		
Comorbidity					
Prior myocardial infarction				8.975	0.003
No	317 (76)	290 (79)	27 (59)		
Yes	98 (24)	79 (21)	19 (41)		
Hypertension, *n* (%)				0.449	0.503
No	238 (57)	209 (57)	29 (63)		
Yes	177 (43)	160 (43)	17 (37)		
Diabetes with complication, *n* (%)				Fisher	1.000
No	400 (96)	355 (96)	45 (98)		
Yes	15 (4)	14 (4)	1 (2)		
Diabetes, *n* (%)				1.628	0.202
No	296 (71)	259 (70)	37 (80)		
Yes	119 (29)	110 (30)	9 (20)		
Hyperlipidemia, *n* (%)				3.713	0.054
No	313 (75)	273 (74)	40 (87)		
Yes	102 (25)	96(26)	6 (13)		
Cerebrovascular disease, *n* (%)				0.121	0.728
No	363 (87)	324 (88)	39 (85)		
Yes	52 (13)	45 (12)	7 (15)		
Atrial fibrillation, *n* (%)				0.676	0.411
No	355 (86)	318 (86)	37 (80)		
Yes	60 (14)	51 (14)	9 (20)		
COPD, *n* (%)				Fisher	0.606
No	407 (98)	361 (98)	46 (100)		
Yes	8 (2)	8 (2)	0 (0)		
Organ or systemic injury					
Acute respiratory failure, *n* (%)				12.563	< 0.001
No	206 (50)	195 (53)	11 (24)		
Yes	209 (50)	174 (47)	35 (76)		
Kidney failure, *n* (%)				18.925	< 0.001
No	220 (53)	210 (57)	10 (22)		
Yes	195 (47)	159 (43)	36 (78)		
Acute liver injury, *n* (%)				25.802	< 0.001
No	318 (77)	297 (80)	21 (46)		
Yes	97 (23)	72 (20)	25 (54)		
Coagulation disorder,*n* (%)				21.951	< 0.001
No	342 (82)	316 (86)	26 (57)		
Yes	73 (18)	53 (14)	20 (43)		
Cardiac insufficiency, *n* (%)				6.127	0.013
No	325 (78)	296 (80)	29 (63)		
Yes	90 (22)	73 (20)	17 (37)		
Neurological injury, n (%)				Fisher	0.126
No	385 (93)	345 (94)	40 (87)		
Yes	30 (7)	24 (6)	6 (13)		
Infection site					
Urinary tract, *n* (%)				4.822	0.028
No	345 (83)	301 (82)	44 (96)		
Yes	70 (17)	68 (18)	2 (4)		
Lung, *n* (%)				2.445	0.118
No	212 (51)	194 (53)	18 (39)		
Yes	203 (49)	175 (47)	28 (61)		
Abdominal cavity, *n* (%)				0.512	0.474
No	283 (68)	249 (67)	34 (74)		
Yes	132 (32)	120 (33)	12 (26)		
Blood culture results					
Klebsiella pneumoniae, *n* (%)				Fisher	0.757
No	386 (93)	342 (93)	44 (96)		
Yes	29 (7)	27 (7)	2 (4)		
Pseudomonas aeruginosa, *n* (%)				Fisher	0.446
No	410 (99)	365 (99)	45 (98)		
Yes	5 (1)	4 (1)	1 (2)		
Escherichia coli, *n* (%)				Fisher	0.106
No	375 (90)	330 (89)	45 (98)		
Yes	40 (10)	39 (11)	1 (2)		
Staphylococcus, *n* (%)				Fisher	1.000
No	380 (92)	338 (92)	42 (91)		
Yes	35 (8)	31 (8)	4 (9)		
Enterococcus, *n* (%)				Fisher	0.219
No	407 (98)	363 (98)	44 (96)		
Yes	8 (2)	6 (2)	2 (4)		
Bowman acinetobacter, *n* (%)				Fisher	0.137
No	403 (97)	360 (98)	43 (93)		
Yes	12 (3)	9 (2)	3 (7)		
Multidrug resistant bacteria				Fisher	0.021
No	388 (93)	349 (95)	39 (85)		
Yes	27 (7)	20 (5)	7 (15)		
Severity score					
SOFA	9 (6, 12)	9 (6, 12)	10 (7.25, 12)	7102.5	0.070
SAPS-II	45 (39, 51)	45 (39, 51)	45.5 (38.25, 50.75)	8502	0.985
Complications					
Cardiopulmonary arrest, *n* (%)				Fisher	0.513
No	389 (94)	347 (94)	42 (91)		
Yes	26 (6)	22 (6)	4 (9)		
Septic shock, *n* (%)				7.247	0.007
No	343(83)	312 (85)	31 (67)		
Yes	72 (17)	57 (15)	15 (33)		

*Abbreviations:* SI-MODS: Sepsis-induced multiple organ dysfunction syndrome; COPD: Chronic obstructive pulmonary disease; SOFA: Sequential organ failure assessment; SAPS-II: Simplified acute physiology score II.

Patients in the SI-MODS group exhibited a higher incidence of anticoagulant use, mechanical ventilation, previous myocardial infarction, acute respiratory failure, kidney failure, acute liver injury, coagulation disorders, cardiac insufficiency, and urinary tract infections compared to those in the non-SI-MODS group. Initial laboratory values such as international normalized ratio (INR), lactate (LAC), total bilirubin, NT-proBNP, alanine aminotransferase (ALT), aspartate aminotransferase (AST), and lactate dehydrogenase (LDH) were significantly higher in septic patients with SI-MODS than in those without (p<0.05). Conversely, fibrinogen (FIB) levels were lower in the SI-MODS group compared to the non-SI-MODS group (p<0.05).

### Predictors entering the model:

**Fig.2:**
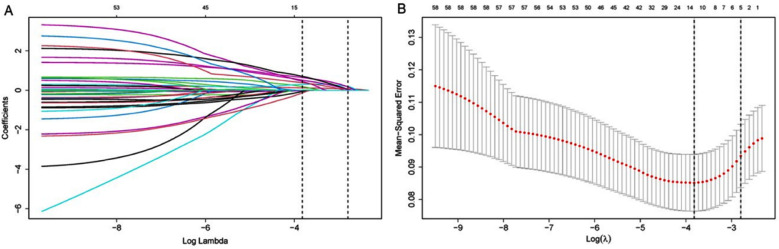
LASSO regression analysis was performed to select the predictors.

LASSO regression analysis was utilized to refine the patient dataset from 62 variables to 14, based on non-zero coefficients (Fig.2A-2B). Using the “Backward: Wald” multiple logistic regression model, seven predictors significantly associated with SI-MODS were identified: partial pressure of oxygen (PaO_2_), LAC, multidrug-resistant bacteria, septic shock, coagulation disorder, mechanical ventilation, and kidney failure (Table-IV).

**Table-IV:** Multivariate logistic regression analysis of predictors selected by LASSO regression procedure.

Independent variables	B	SE	OR	95% CI		Z	p-Value
				Lower	Upper		
PaO_2_	-0.011	0.005	0.989	0.978	0.999	-1.991	0.046
LAC	0.178	0.040	1.195	1.105	1.295	4.419	<0.001
Multidrug resistant bacteria	1.398	0.569	4.047	1.274	12.120	2.458	0.014
Septic shock	0.622	0.404	1.863	0.827	4.066	1.541	0.123
Coagulation disorder	1.156	0.394	3.177	1.458	6.883	2.936	0.003
Mechanical ventilation	2.280	0.784	9.780	2.643	65.760	2.908	0.004
Kidney failure	1.150	0.413	3.158	1.446	7.413	2.784	0.005

*Abbreviations:* OR: Odds ratio; CI: Confidence interval; SE: Standard error; PaO_2_: Arterial partial pressure of oxygen; LAC: Lactate.

### Establishment of the Model and Nomogram:

A predictive model for SI-MODS was developed based on seven crucial predictors, and the coefficients assigned to each predictor in the model were as follows: PaO_2_ (-0.011), LAC (0.178), Multidrug-resistant bacteria (1.398), Septic shock (0.622), Coagulation disorder (1.156), Mechanical ventilation (2.280), and Kidney failure (1.150).

**Fig.3:**
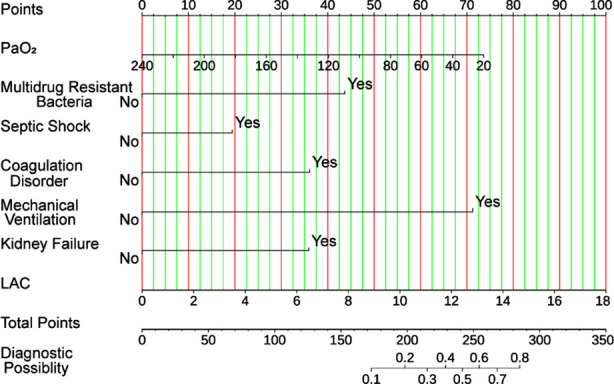
The predictive nomogram can evaluate the risk of SI-MODS in patients with sepsis. ***Abbreviations:*** PaO_2_: Arterial partial pressure of oxygen; LAC: Lactate.

A nomogram was constructed to assess the individual risks of SI-MODS using multivariate logistic regression analysis (Fig.3). The model’s derived formula was: logistic (risk score) = -5.162 -0.011 × PaO_2_ + 0.178 × LAC + 1.398 × Multidrug resistant bacteria + 0.622 × Septic shock + 1.156 × Coagulation disorder + 2.280 × Mechanical ventilation + 1.150 × Kidney failure (Table-III).

**Fig.4:**
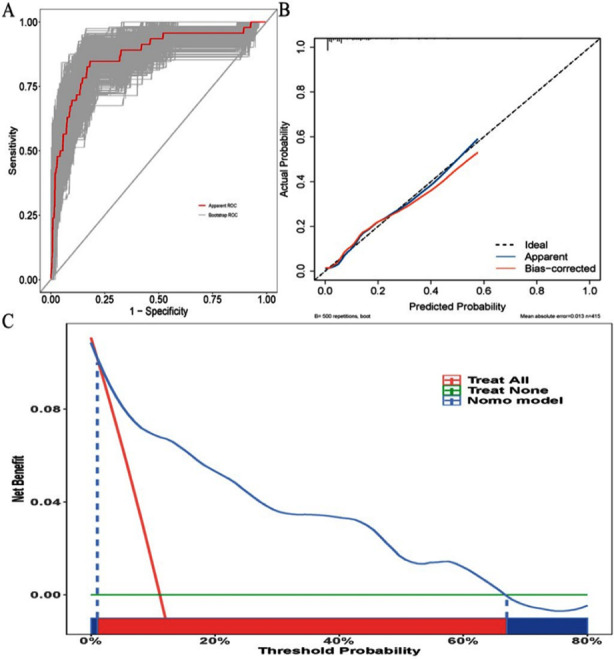
The nomogram model was verified through the following analyses. **(A)** The nomogram-related ROC curve was constructed by bootstrap resampling (500 iterations). **(B)** The predictive accuracy of the nomogram was evaluated using a calibration plot. **(C)** DCA was performed for the nomogram, and it illustrates the expected net benefit per patient based on the nomogram’s prediction of SI-MODS risk.

To evaluate the accuracy of the nomogram, multiple methodologies were employed. The area under the curve (AUC) of the nomogram was 0.903 (95% CI: 0.863-0.943), as determined through a bootstrap method involving 500 resamples (Fig.4A). The predicted probabilities from the nomogram showed a high concordance with observed clinical outcomes, as depicted in Fig.4B. Additionally, a Hosmer-Lemeshow test yielded a p-Value of 0.290, indicating excellent model fit. In the decision curve analysis (DCA), the nomogram’s risk estimates for SI-MODS demonstrated a significantly greater net benefit compared to both the “screen-none” and “screen-all” strategies, particularly when the threshold probability for SI-MODS ranged from 1% to 66% (Fig.4C).

### Model comparison:

**Supplementary Fig.1:**
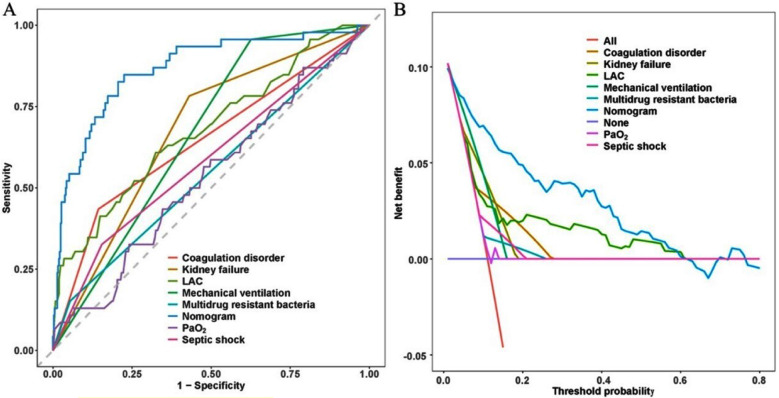
Comparison of models in the entire study cohort. (A) ROC curves of various models. (B) DCA curves of various models. ***Abbreviations:*** PaO_2_: Arterial partial pressure of oxygen; LAC: Lactate.

Supplementary Fig.1A presented the results of the receiver operating characteristic (ROC) curve analysis. The AUC values were as follows: 0.903 for the nomogram, and 0.535, 0.679, 0.549, 0.586, 0.646, 0.665, and 0.676 for PaO_2_, LAC, multidrug-resistant bacteria, septic shock, coagulation disorder, mechanical ventilation, and kidney failure, respectively. Notably, the nomogram demonstrated superior discriminatory accuracy in identifying patients at risk of SI-MODS compared to each individual variable within the nomogram (all *P* < 0.05).

The clinical utility of the model was assessed using DCA. The results revealed that the nomogram outperformed models that solely incorporated risk factors from within the nomogram itself. This advantage was evident in the overall net benefit across a broad spectrum of threshold probabilities (Supplementary Fig.1B).

**Supplementary Fig.2:**
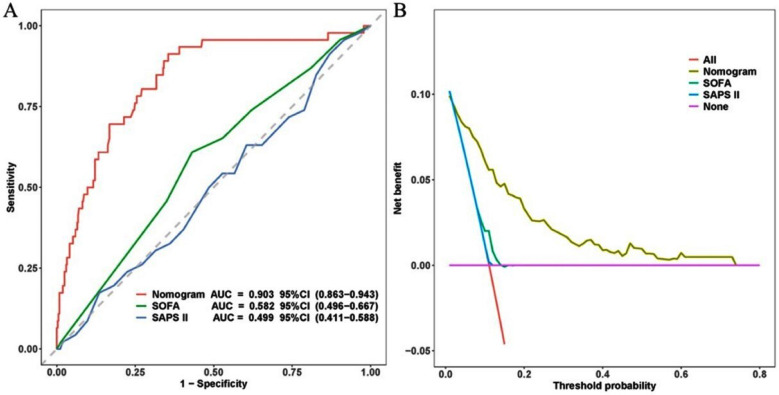
Comparison of ROC and DCA curves between the predictive model (nomogram) and the SOFA and SAPS-II scoring methods. (A) ROC curves; (B) DCA curves. ***Abbreviations:*** SOFA: Sequential organ failure assessment; SAPS-II: Simplified acute physiology score II; AUC: Area under curve; CI: Confidence interval.

The predictive model demonstrated superior performance compared to both the SOFA and SAPS-II in assessing the risk of SI-MODS in sepsis patients, with statistically significant differences (both *P* < 0.05, Supplementary Fig.2A). The AUC for the predictive model was 0.903 (95% CI: 0.863-0.943), significantly higher than the AUCs for the SOFA score (0.582, 95% CI: 0.496–0.667) and the SAPS-II score (0.499, 95% CI: 0.411–0.588).

DCA was conducted to evaluate the clinical utility of the predictive model and conventional scoring methods. The analysis demonstrated that for threshold probabilities greater than 1%, interventions based on the nomogram yielded a higher net benefit than those guided by the SOFA and SAPS-II (Supplementary Fig.2B).

## DISCUSSION

In the present study, we firstly constructed a nomogram to predict SI-MODS, utilizing seven variables from sepsis patients. These variables included PaO_2_, LAC, multidrug resistant bacteria, septic shock, coagulation disorder, mechanical ventilation, and kidney failure. The nomogram showed good discriminatory ability, calibration, and clinical usefulness, which is considered more effective than univariate analysis for choosing predictors.^13^,^14^ Consistently, Luo et al, developed a nomogram based on age, diastolic blood pressure, LAC, PaO_2_, platelet, mechanical ventilation, and found that it could help clinicians reasonably determine the risk of sepsis associated-acute respiratory failure.^15^ Xu et al, established a nomogram incorporating thirteen clinical features including septic shock, and showed a predictive ability to predict the acute respiratory distress syndrome risk in patients with sepsis.^16^ In addition, it has been reported that the emergence of multidrug-resistant organisms (MDROs) has intensified the challenges in managing multidrug-resistant sepsis.^17^ Coagulation disorder is closely associated with SI-MODS, and anticoagulant therapy is considered as an effective treatment for SI-MODS.^18^ Moreover, kidney failure is one of the most frequent complications underlying sepsis.^19^

Besides, this predictive model also demonstrated superior performance compared to both the SOFA and SAPS-II in assessing the risk of SI-MODS in sepsis patients. The AUC for the predictive model was 0.903 (95% CI: 0.863-0.943), significantly higher than the AUCs for the SOFA score (0.582, 95% CI: 0.496-0.667) and the SAPS-II score (0.499, 95% CI: 0.411-0.588). DCA analysis further proved the clinical utility of the predictive model, and demonstrated that for threshold probabilities greater than 1%, interventions based on the nomogram yielded a higher net benefit than those guided by the SOFA and SAPS-II. Therefore, the combined model may be a better choice for predicting MODS in sepsis patients, which was in line with previous reports.^20^,^21^

### Limitations:

This predictive model presents several limitations that warrant attention. Firstly, the nomogram was developed from data collected during a three-year prospective study at the First Affiliated Hospital of Nanjing Medical University in Huai’an. Consequently, further multicenter studies are essential to validate the nomogram’s applicability across different regions or countries. Secondly, the nomogram does not account for the etiology of SI-MODS. This limitation signifies an area of uncertainty that necessitates further research to understand how SI-MODS develops. Thirdly, the analysis excluded certain patient variables, such as treatment regimens and duration, due to their absence in the initial dataset. The inclusion of these variables in future studies could potentially improve the predictive accuracy of the model.

## CONCLUSION

Our study firstly develops a nomogram incorporating based on PaO_2_, LAC, multidrug resistant bacteria, septic shock, coagulation disorder, mechanical ventilation, and kidney failure can predict the risk of MODS in sepsis patients, which helps clinicians make risk-based decisions and treatment strategies.

### Authors’ Contributions:

**JJ and QW:** Conceived, designed the study and were involved in writing and revising of the manuscript.

**KW, TS and CL:** Collected the data and performed the analysis.

All authors have read, approved the final manuscript and are responsible for the integrity of the study.
